# Retraction notice to "Applying analytic hierarchy process and failure likelihood index method (AHP-FLIM) to assess human reliability in critical and sensitive jobs of a petrochemical industry" [Heliyon 8 (2022) e09509]

**DOI:** 10.1016/j.heliyon.2025.e43555

**Published:** 2025-06-28

**Authors:** Asma Zare, Naser Hoboubi, Salman Farahbakhsh, Mehdi Jahangiri

**Affiliations:** a Department of Occupational Health Engineering, Sirjan School of Medical Sciences, Sirjan, Iran; b Student Research Committee, Department of Occupational Health Engineering, Shiraz University of Medical Sciences, Shiraz, Iran; c Research Center for Health Sciences, Department of Occupational Health Engineering, School of Health, Shiraz University of Medical Sciences, Shiraz, Iran

This article has been retracted: please see Elsevier policy on article withdrawal (https://www.elsevier.com/about/policies-and-standards/article-withdrawal).

This article has been retracted at the request of the Editor.

A post-publication investigation conducted by Elsevier's Research Integrity & Publishing Ethics team on behalf of the journal could not confirm whether or not ethical approval was given and whether or not the participants provided informed consent for the research presented in this publication in line with the journal's policy. As it cannot be established whether the article complies with the journal's policies on ethical approval and consent, the editor has decided to retract the article.

The authors have not responded to the retraction notice.

